# A Novel Liposome Formulation Carrying Both an Insulin Peptide and a Ligand for Invariant Natural Killer T Cells Induces Accumulation of Regulatory T Cells to Islets in Nonobese Diabetic Mice

**DOI:** 10.1155/2019/9430473

**Published:** 2019-10-23

**Authors:** Hidetoshi Akimoto, Emi Fukuda-Kawaguchi, Omar Duramad, Yasuyuki Ishii, Kazunari Tanabe

**Affiliations:** ^1^Research Division, REGiMMUNE Corporation, 35-3 Nihonbashi Hakozaki-cho, BRICK GATE 5F, Chuou-Ku, Tokyo 103-0015, Japan; ^2^Research Division, REGiMMUNE Inc, 820 Heinz Ave, Berkeley, CA 94710, USA; ^3^Department of Urology, Tokyo Women's Medical University, 8-1 Kawada-cho, Shinjuku-Ku, Tokyo 162-8666, Japan

## Abstract

Type 1 diabetes (T1D) is an autoimmune disease caused by the destruction of pancreatic *β* cells by autoantigen-reactive diabetogenic cells. Antigen-specific therapies using islet autoantigens for restoring immune tolerance have emerged as promising approaches for the treatment of T1D but have been unsuccessful in humans. Herein, we report that RGI-3100-iB, a novel liposomal formulation carrying both *α*-galactosylceramide (*α*-GalCer), which is a representative ligand for invariant natural killer T (iNKT) cells, and insulin B chain 9–23 peptide, which is an epitope for CD4^+^ T cells, could induce the accumulation of regulatory T cells (Tregs) in islets in a peptide-dependent manner, followed by the remarkable prevention of diabetes onset in nonobese diabetic (NOD) mice. While multiple administrations of a monotherapy using either *α*-GalCer or insulin B peptide in a liposomal formulation was confirmed to delay/prevent T1D in NOD mice, RGI-3100-iB synergistically enhanced the prevention effect of each monotherapy and alleviated insulitis in NOD mice. Immunopathological analysis showed that Foxp3^+^ Tregs accumulated in the islets in RGI-3100-iB-treated mice. Cotransfer of diabetogenic T cells and splenocytes of NOD mice treated with RGI-3100-iB, but not liposomal *α*-GalCer encapsulating an unrelated peptide, to NOD-SCID mice resulted in the prevention of diabetes and elevation of Foxp3 mRNA expression in the islets. These data indicate that the migration of insulin B-peptide-specific Tregs to islet of NOD mice that are involved in the suppression of pathogenic T cells related to diabetes onset and progression could be enhanced by the administration of liposomes containing *α*-GalCer and insulin B peptide.

## 1. Introduction

Type 1 diabetes (T1D) is an autoimmune disease characterized by progressive destruction of pancreatic *β* cells by diabetogenic CD4^+^ and CD8^+^ T cells and B cells, which have loss of immunological tolerance and react with defined islet proteins, such as insulin [1]. Currently, patients with T1D require lifelong standard “insulin therapy” with daily administration of insulin injection and monitoring of blood glucose levels (BGLs). However, even while maintaining healthy BGLs, patients remain at risk of acute and late complications, including ischemic heart disease, retinopathy, and nephropathy [2, 3]. As alternative treatments, an antigen-nonspecific therapy (using monoclonal antibodies against T/B cells and low-dose IL-2 and cellular therapies) and an antigen-specific therapy (an autoantigen-based immunotherapy using insulin, insulin B peptide, proinsulin, or GAD) have been tested in clinical trials [4–7], but unfortunately, there is no cure for T1D, as of date [8]. If an antigen-specific therapy is effective, the treatment could stop further destruction of insulin-producing cells by restoring immune tolerance without impairing the normal immunity; therefore, it is recognized as a primary therapy for preventing the onset or reversal of T1D.

Regulatory T cells (Tregs) are one of the targets of an immunotherapy. They play a critical role in the maintenance of immune homeostasis and autoimmunity [9]. The manipulation of Tregs, especially autoantigen-specific Tregs, is considered a promising treatment for T1D [10] because it has a high potential to restore immune tolerance by antigen-dependent accumulation in draining lymph nodes and inflamed islets [11] and local bystander suppression of other islet-infiltrating pathogenic cells [12], without impairing beneficial immune responses. Indeed, a number of studies have shown that autoantigen-specific Treg cells are able to prevent the development of T1D as well as reverse the established diabetes in rodent models [13–16]: Insulin B chain 9–23 peptide is known to induce Treg cells [17] that produce TGF-beta [18, 19] in NOD mice to prevent T1D. We have previously reported that a novel liposomal formulation carrying an antigenic polypeptide and *α*-galactosylceramide (*α*-GalCer), an activator of invariant natural killer T (iNKT) cells, induced the antigen-specific Tregs *in vivo*, and the suppression of both primary and secondary antibody responses in preclinical mouse models of allergy [20, 21].

iNKT cells are well known to have a strong potential in preventing and reversing T1D [22–24]. These cells are nonconventional T lymphocytes, expressing a single invariant antigen receptor encoded by V*α*14J*α*18 in the mouse and V*α*24J*α*18 in humans [25–27], and are restricted by the major histocompatibility complex (MHC) class I-like CD1d molecule [28]. The CD1d is a nonpolymorphic, antigen-presenting molecule expressed on professional antigen-presenting cells (APCs), with an antigen-binding groove adapted for the presentation of glycolipid antigens, such as *α*-galactosylceramide (*α*-GalCer, originally discovered from a marine sponge) [29]. Interestingly, repetitive treatment of aqueous *α*-GalCer (in DMSO- and detergent-dissolved aqueous forms) was shown to prevent and stop the recurrence of spontaneous diabetes development in nonobese diabetic (NOD) mice; however, there was no enhancement of the regulatory activity of spleen CD62L^+^ regulatory T cells toward diabetogenic T cells [23]. It was suggested that simultaneous stimulation of naïve T cells with regulatory iNKT cells and antigen presentation of APCs might be essential for the induction and enhancement of antigen-specific immune tolerance.

Based on the above hypothesis, attempts were made to generate a novel product of liposomal *α*-GalCer encapsulating insulin B 9–23 epitope peptide (RGI-3100-iB). The treatment of prediabetic NOD mice with RGI-3100-iB prevented T1D development, diminished insulitis, and resulted in the accumulation of Foxp3^+^ Tregs in an antigen-specific manner. Furthermore, the treatment with RGI-3100-iB led to the synergistic enhancement of the suppression activity of regulatory cells against diabetogenic cells and in increased levels of Foxp3 mRNA in the islets. This approach might prevent T1D through autoantigen-specific reestablishment of immune tolerance.

## 2. Materials and Methods

### 2.1. Reagents

KRN7000, a representative *α*-galactosylceramide (*α*-GalCer) compound, was synthesized by REGiMMUNE Corp. (Japan). Insulin peptide B (9–23) and tetanus toxin (830–844) were purchased from AnaSpec Inc. (Fremont, CA). Dioleoylphosphatidylcholine (DOPC), 1,2-dioleoylphosphatidylglycerol (DOPG), and cholesterol were purchased from Avanti Polar Lipids Inc. (Alabaster, AL). Anti-CD3 antibody (clone 145-2C11) was purchased from Bio X cell (West Lebanon, NH) and was used as a positive control for preventive treatment of diabetes.

### 2.2. Mice

For assessment of diabetes, female NOD/ShiLtJ mice obtained from The Jackson Laboratory (Bar Harbor, ME) were used. For evaluation of insulitis and for adoptive transfer study, NOD/ShiJcl and NOD-SCID mice obtained from CLEA Japan Inc. (Tokyo, Japan) were used. All the mice were maintained under specific pathogen-free conditions, housed in autoclaved cages, and provided with autoclaved food and water. The studies conducted at The Jackson Laboratory were performed according to an IACUC-approved protocol and in compliance with the Guide for the Care and Use of Laboratory Animals. The experiments conducted at Tokyo Women's Medical University were performed according to a protocol approved by internal and external committees and were in compliance with the prescribed guidelines.

### 2.3. Preparation of Liposomes and Treatments

For all formulations, the lipids, DOPC, DOPG, and Chol, were used at a molar ratio of 50 : 20 : 30. For preparation of liposomes containing *α*-GalCer, phospholipids and cholesterol (10 mg) and *α*-GalCer (0.2 mg) were dissolved in a mixture of chloroform and methanol (1 : 1). They were then combined in glass tubes and dried to a thin film by rotary evaporation under reduced pressure. For normal liposomes, *α*-GalCer was not added. The dried films were suspended in 300 *μ*L of 50 mM Tris HCl buffer (pH 8.5), containing 1 mg of insulin B peptide, or in distilled water containing 1 mg of tetanus toxin peptide, and homogenized by vortexing and sonication. After five cycles of freeze-thaw using liquid nitrogen and heat block, 700 *μ*L of buffer was added to make up the volume of 1 mL. Unilamellar liposomes were prepared from multilamellar liposome suspensions using a LiposoFast-Basic extruder (Avestin Inc., Ottawa, ON, Canada) by 25 cycles of manual extrusion through a 100-nm-pore-size polycarbonate membrane. Loss of *α*-GalCer and lipids trapped in the extruder was less than 10% of the starting material, as estimated by the weight of lipids recovered. To remove free peptide, the liposomal dispersion was filled in a dialysis membrane (cutoff = 1000 kDa) and dialyzed for 72 h at 4°C against a total of 9 L buffer to allow the free peptide to diffuse out. The lipid concentration was determined by LabAssay™ Phospholipid (FUJIFILM Wako, Osaka, Japan). The peptide concentration was determined by BCA protein assay (Thermo Fisher Scientific, Waltham, MA). The vesicle size, polydispersity index, and zeta potential of liposomes were determined by dynamic light scattering (Malvern Instruments, Malvern, UK). Liposomes were stored at 4°C under argon. NOD mice were administered intraperitoneal injections of each liposome (2 *μ*g *α*-GalCer and/or 2 *μ*g peptide/injection) starting at 3 or 4 weeks of age, twice a week, for a period of 5 weeks (total 10 injections). As a positive control, a single injection of anti-CD3 antibody was given to mice on postnatal day 7.

### 2.4. Assessment of Diabetes Incidence and Evaluation of Insulitis

Diabetes was assessed by monitoring glucose levels every week in the blood using blood glucose Accu-Check Aviva Plus test strips (Roche, Indianapolis, IL) and an Accu-Check blood glucose meter (Roche). Mice were designated as diabetic when blood glucose levels were greater than 250 mg/dL for two consecutive weeks.

### 2.5. Histopathology of NOD Pancreata

For evaluation of insulitis, pancreatic specimens of NOD mice were fixed with 10% formalin, embedded in Tissue-Tek OCT compound (Sakura Finetek, Torrance, CA), and frozen at –70°C, and 5 *μ*m cryosections were made. Serial sections were stained with hematoxylin and eosin (H&E) for visualization of general morphology to evaluate insulitis. Multiple H&E-stained pancreatic sections were scored in a blinded fashion [30, 31]. Pancreatic islets were assigned scores as follows: 0, intact islets/no lesions; 1, peri-islet infiltrates; 2, <25% islet destruction; 3, >25% islet destruction; and 4, complete islet destruction.

### 2.6. Immunohistochemistry of Foxp3^+^ Cells in Islets

For identification of Tregs, 5 *μ*m cryosections from pancreatic specimens of NOD mice were fixed in ice-cold acetone and subjected to methanol blocking and biotin/avidin-blocking (Vector Laboratories, Burlingame, CA). Slides were then incubated with biotinylated anti-Foxp3 mAb (eBioscience, Thermo Fisher Scientific). Because Foxp3 is a low-abundant target, signal was amplified using HRP-streptavidin and Alexa Fluor 488 tyramide reagent (Invitrogen, Thermo Fisher Scientific), according to the manufacturer's instructions. Fluorescent images were obtained with a fluorescence microscope, Biozero (Keyence, Osaka, Japan).

### 2.7. Adoptive Transfer of Diabetes into NOD-SCID Mice

To test the effect of different treatments on the regulatory activity of regulatory T cells in NOD mice, cotransfer of diabetogenic T cells into NOD-SCID mice was assessed. Diabetogenic cells were recovered by gentle disruption of spleen retrieved from at least three recently overtly diabetic NOD females. As already reported by several researchers, diabetogenic T cells exclusively comprise the CD62L^–^ T cell population [32, 33]; therefore, the cell suspension was stained for CD4, CD8, and CD62L to determine the percentage of CD62L^–^ T cells. To normalize diabetes transfers, the number of spleen cells injected was calculated so that each recipient received 4 × 10^5^ CD62L^–^ T cells (corresponding to 1.2 × 10^6^T cells or 3 × 10^6^ spleen cells) intravenously. To test the regulatory function of total spleen cells, pooled splenocytes (40 × 10^6^) from prediabetic 10-week-old female NOD mice (6 mice/group), one week after the last treatment of test drugs from 4 to 8 weeks, twice a week, were prepared and cotransferred into 5-week-old female NOD-SCID hosts with diabetogenic T cells. The recipients (3 mice/group) were analyzed weekly for BGL.

### 2.8. Isolation of Islets and Analysis of Foxp3 mRNA Expression in T Cells

Each pancreas sample was perfused with a solution of collagenase P (1 mg/mL, Sigma–Aldrich, Merck), dissected, and incubated at 37°C for 15 min. Islets were purified using a Histopaque (Sigma–Aldrich, Merck) gradient and were handpicked and counted. Total RNA was extracted from the isolated islets using NucleoSpin RNA (TaKaRa Bio Inc., Shiga, Japan), and cDNA was produced using Superscript III reverse transcriptase (Invitrogen, Thermo Fisher Scientific). Islets isolated from three mice were pooled for each sample to reduce interindividual variability in the RNA expression analyses. Quantitative-PCR was carried out using TaqMan Gene Expression Assays (Applied Biosystems, Thermo Fisher Scientific). CD3 was used as a reference to normalize all the samples.

### 2.9. Statistical Analysis

A log-rank test was applied to compare the diabetes incidence. Student's *t*-test was used to calculate the statistical significance, wherever indicated. *P*-values smaller than 0.05 were considered to be statistically significant.

## 3. Results

### 3.1. Treatment with RGI-3100-iB Prevents Spontaneous T1D Development in NOD Mice

**Figure 1:**
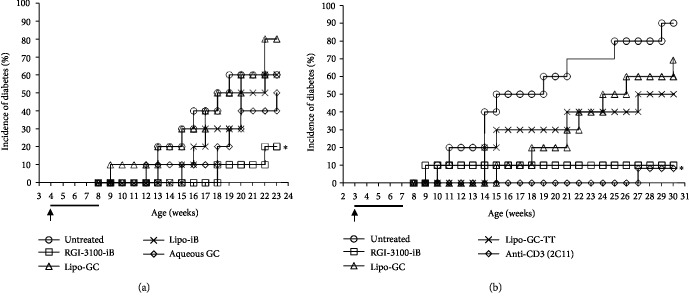
Suppression of spontaneous diabetes development in nonobese diabetic (NOD) mice after administration of autoantigen-loaded *α*-GalCer liposomes. (a) Diabetes in female NOD mice intraperitoneally injected with insulin B 9–23-loaded *α*-GalCer liposome (RGI-3100-iB: □; *n* = 10), *α*-GalCer liposome (Lipo-GC: △; *n* = 10), insulin B 9–23-loaded normal liposome (Lipo-iB: ×; *n* = 10), and aqueous *α*-GalCer (aqueous GC: ◇; *n* = 10), twice a week, starting at 4 weeks of age, for 5 weeks, or left untreated (○; *n* = 10). (b) Diabetes in female NOD mice intraperitoneally injected with insulin B 9–23-loaded *α*-GalCer liposome (RGI-3100-iB: □; *n* = 10), *α*-GalCer liposome (Lipo-GC: △; *n* = 10), and unrelated antigen-loaded *α*-GalCer liposome (Lipo-GC-TT: ×; *n* = 10), twice a week, starting at 3 weeks of age, for 5 weeks, or left untreated (○; *n* = 10). Anti-CD3 antibody (2C11) (◇; *n* = 10) was administered, as a positive control, to mice at postnatal day 7. Arrows and black thick bars indicate the initiation and duration of the treatment, respectively. ∗*P* < 0.05 log-rank test, compared with untreated mice.

To investigate the effects of codelivery of *α*-GalCer and autoantigen on the development of diabetes in NOD mice, four different types of liposomes were prepared: (1) a liposomal *α*-GalCer encapsulating insulin B 9–23 peptide (RGI-3100-iB); (2) a liposomal *α*-GalCer without antigen (lipo-GC); (3) a liposome encapsulating insulin B peptide, without *α*-GalCer (lipo-iB); and (4), a liposomal *α*-GalCer encapsulating an unrelated antigen, tetanus toxin peptide (lipo-GC-TT). To evaluate the effect of RGI-3100-iB on the development of diabetes, two independent *in vivo* experiments were conducted using NOD mice. In one experiment, all the test substances were injected twice a week from 4 to 8 weeks of age. The onset of diabetes at 23 weeks of age in NOD mice was significantly prevented by the RGI-3100-iB treatment (2/10, 20% incidence) compared with that in the untreated mice (6/10, 60%) (Figure 1(a)). The onset was not fully prevented in other treatments with lipo-GC (8/10, 80%), lipo-iB (6/10, 60%), and aqueous *α*-GalCer (5/10, 50%). We performed another experiment to confirm the significance of antigen specificity in the RGI-3100-iB treatment. All the test substances, except anti-CD3 mAb, were injected twice a week from 3 to 7 weeks of age. The onset of diabetes at 30 weeks of age in NOD mice was significantly prevented by the RGI-3100-iB treatment (1/10, 10%), but not by the lipo-GC-TT peptide (5/10, 50%) when compared with the onset in the untreated mice (8/10, 80%) (Figure 1(b)). Notably, the protective effect of RGI-3100-iB treatment was mostly comparable with that of anti-CD3 treatment at postnatal day 7 (1/11, 9%). These results indicated that the combination treatment with *α*-GalCer and insulin B peptide 9–23 in RGI-3100-iB could synergistically enhance the prevention of spontaneous diabetes development in NOD mice.

### 3.2. Treatment with RGI-3100-iB Diminishes Insulitis in NOD Mice

**Figure 2:**
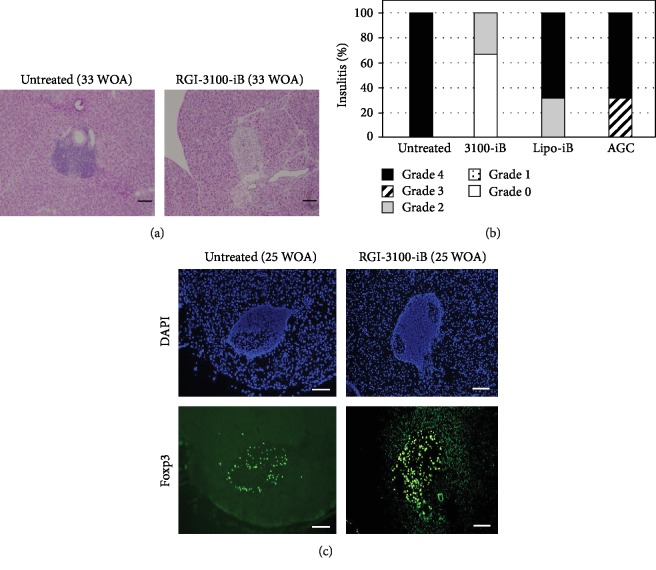
Histopathological evaluation of insulitis and immunohistochemistry of Foxp3^+^ cells in islets. (a) Representative insulitis formation visualized in hematoxylin and eosin-stained pancreas sections from 33-week-of-age (WOA) nonobese diabetic (NOD) mice subjected to the indicated treatments (25 weeks after the last drug treatment). (b) Percentage of islets with a given degree of infiltration at 33 WOA in NOD female mice subjected to the indicated treatments. (c) Representative Foxp3^+^ cell accumulation in Foxp3-stained pancreatic islet sections from 25 WOA NOD mice subjected to the indicated treatments. DAPI (blue) stains for nuclei. Data were obtained from three independent experiments. Bars: 100 *μ*m.

To confirm the correlation between the incidence of diabetes and insulitis, histological analysis of sections of pancreatic islets was done in treated mice. The insulitis was clearly diminished in RGI-3100-iB-treated NOD mice compared with nontreated or control liposome-treated NOD mice, indicating a positive correlation between the preventive effect and reduction of insulitis (Figures 2(a) and 2(b)). Because it has been reported that antigen-specific Tregs have a property to accumulate around inflamed target tissues [11], attempts were made to observe Tregs in pancreatic islets of RGI-3100-iB-treated NOD mice. By immunohistochemical staining of Foxp3 in cells, a large number of Foxp3^+^ cells in the islets of RGI-3100-iB-treated 25-week-old NOD mice were observed than in those of nontreated NOD mice (Figure 2(c)). Overall, these results suggest that Foxp3^+^ cells accumulating in the islets upon treatment with RGI-3100-iB might play a protective role against the progression of insulitis in NOD mice.

### 3.3. Adoptive Transfer of Splenic T Cells from RGI-3100-iB-Treated NOD Mice Delays the Development of Diabetes in NOD-SCID Mice

**Figure 3:**
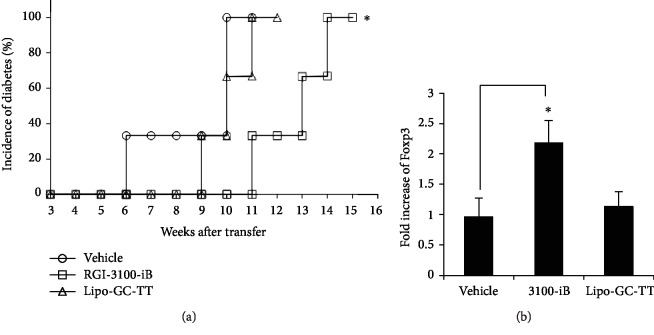
Autoantigen-loaded *α*-GalCer liposome treatment enhances the protective activity of regulatory cells and increases Foxp3^+^ mRNA expression in islets. (a) Diabetogenic CD62L^–^ T cells were cotransferred into three NOD-SCID recipients, and splenocytes were collected from NOD mice 1 week after the last injection of the following treatments: RGI-3100-iB (□; *n* = 6), Lipo-GC-TT (△; *N* = 6), or vehicle (injection○; *n* = 6). (b) qPCR analyses of Foxp3 transcripts in islets collected from NOD mice subjected to the indicated treatments. Data are represented as mean values ± SD obtained in three independent experiments, each performed with three pooled mice. ∗*P* < 0.05 Student's *t*-test, compared with the vehicle treatment.

To test whether the adoptive transfer of splenocytes treated with RGI-3100-iB could reproduce the effects upon *in vivo* treatment, diabetogenic T cells were injected together with splenocytes from the treated prediabetic mice into NOD-SCID host mice. To prepare the splenocytes from the treated prediabetic NOD mice, RGI-3100-iB, vehicle, or lipo-GC-TT was administered intraperitoneally into NOD mice starting at 4 weeks of age, twice a week, for 5 weeks. The cotransfer of splenocytes from RGI-3100-iB-treated NOD mice clearly showed the reproducibility of the preventive efficacy, with delayed onset of adoptively transferred T1D in NOD-SCID mice compared with that in the case of cotransfer of splenocytes from vehicle- or lipo-GC-TT-treated NOD mice (Figure 3(a)). To confirm whether adoptively transferred Foxp3^+^ cells from NOD mice treated with RGI-3100-iB accumulate in the islets of NOD-SCID mice, the expression levels of Foxp3 mRNA from infiltrates in the islets were analyzed by qPCR. The Foxp3 mRNA expression levels in the infiltrates in NOD-SCID mice infused splenocytes from RGI-3100-iB-treated NOD mice showed over twofold increase compared with those in the infiltrates in NOD-SCID mice treated with vehicle or lipo-GC-TT (Figure 3(b)). This demonstrates the reproducibility of induction of Foxp3^+^ cell accumulation in islets by treatment of NOD mice with RGI-3100-iB (Figure 2(c)). These results suggest that the liposomal *α*-GalCer treatment with insulin B 9–23 peptide might enhance the peptide-dependent accumulation of Foxp3^+^ Tregs in islets, followed by the preventive effect against the spontaneous development of diabetes and protective activity against insulitis progression in NOD mice.

## 4. Discussion

Manipulation of autoantigen-specific Tregs has emerged as an attractive approach for preventing and/or curing autoimmune diseases because it can attenuate autoimmune responses without impairing the beneficial immune responses by targeting autoreactive pathogenic cells [10, 13–16]. In this study, we demonstrate synergistic enhancement of the prevention effect on T1D and alleviation of insulitis in NOD mice by combining an autoantigen and a ligand for iNKT cells in a liposomal formulation (Figures 1, 2(a), and 2(b)). We confirm the results of previous studies by others wherein it has been shown that the insulin B chain 9–23 peptide delays/prevents T1D in NOD mice [17–19] by using lipo-iB (Figure 1(a)) as well as of other studies showing that *α*-GalCer alone delays/prevents T1D [22–24] by using aqueous *α*-GalCer (Figure 1(a)) and lipo-GC-TT (Figure 1(b)). We also show the accumulation of Foxp3^+^ cells in pancreatic islets and enhancement of the protective activity of regulatory cells against diabetogenic cells in the mouse models after the treatment (Figures 2(c) and 3).

As reported in other studies using rodent experiments, antigen delivery to the appropriate APC subsets, followed by initiation of the cascade of tolerogenic signaling pathways, is essential for the induction of antigen-specific immune tolerance [34]. Simultaneous activation of iNKT cells by *α*-GalCer is a promising approach to bring an optimal regulatory milieu to the interaction between T cells and APCs, with antigen presentation by MHC II together with low levels of costimulatory molecule expression and absence of other activating stimuli (i.e., inflammation, infection, or other pathologies) [20, 21, 35, 36]. No attempt has been made to combine iNKT activation using *α*-GalCer and autoantigen presentation to induce immune tolerance for T1D treatment. We successfully prepared liposomal *α*-GalCer, encapsulating the insulin B 9–23 epitope peptide (RGI-3100-iB). Sharif et al. previously reported that aqueous *α*-GalCer treatment for prevention of disease onset in NOD mice failed to enhance the regulatory activity of splenic CD62L^+^ T cells, which could not inhibit the activity of diabetogenic T cells in a cotransfer T1D model using NOD-SCID mice, although its administration prevented the onset and recurrence of diabetes in NOD mice [23]. In contrast, our results using RGI-3100-iB suggest that liposomal *α*-GalCer, encapsulating an autoantigen, might modify the protective activity of regulatory T cells toward diabetogenic T cells in the islets in a peptide-dependent manner (Figure 3). We confirmed the results of previous studies that the insulin B chain 9–23 peptide (lipo-iB; Figure 1(a)) and *α*-GalCer (aqueous *α*-GalCer, Figure 1(a); lipo-GC-TT, Figure 1(b)) alone can delay/prevent T1D.

**Figure 4:**
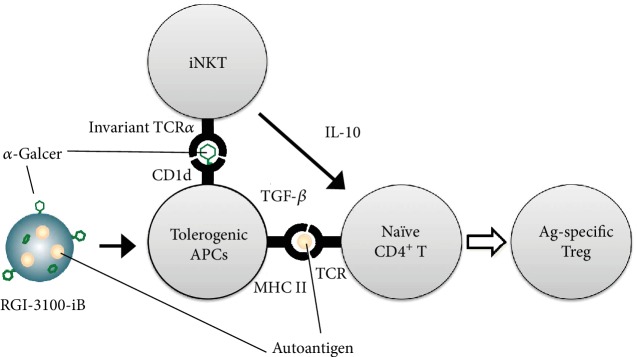
Predicted mode of action of autoantigen-loaded *α*-GalCer liposome in autoantigen-specific Treg induction. RGI-3100-iB delivers autoantigen and *α*-GalCer to antigen-presenting cells (APCs). After several steps of processing of liposome in the late endosome/lysosome, APCs present the antigen via their MHC II and *α*-GalCer via CD1d that activates iNKT cells to secrete the tolerogenic cytokine, IL-10. Under the regulatory environment created by the activated iNKT cells, APCs interact with naïve diabetogenic effector CD4^+^ T cells via specific antigen-loaded MHC II and costimulatory molecules, resulting in the conversion to autoantigen-specific Tregs.

On the basis of a series of studies conducted by us, we expect the mechanism of tolerance-inducing action of RGI-3100-iB in preventing diabetes onset to be as shown in Figure 4. First, it is worth comparing the mode of action of aqueous *α*-GalCer with that of RGI-3100-iB in the treatment of T1D in NOD mice. The aqueous *α*-GalCer treatment led to the induction of tolerogenic dendritic cells via the production of Th2-biased cytokines, such as IL-4 and IL-10, by iNKT cells, resulting in skewing the Th responses and conversion of naïve effector CD4^+^ T cells into polyclonal Tregs, including antigen-specific Tregs together with polyclonal Tregs [22, 23]. In contrast, RGI-3100-iB was developed with the concept to induce antigen-specific immune tolerance based on the finding from our previous allergy model studies [21]. By codelivering autoantigen and *α*-GalCer, APCs present the antigen via their MHC II and *α*-GalCer via CD1d and interact with naïve diabetogenic effector CD4^+^ T cells under the regulatory environment created by activated iNKT cells secreting the tolerogenic cytokine, IL-10, resulting in the conversion to antigen-specific Tregs. Although it remains to be determined as to how the phenotype and function of Tregs are varied by the RGI-3100-iB treatment, as indicated by the accumulation of Foxp3^+^ cells in the islets (Figures 2(c) and 3(b)) but no alteration in the overall cell number and population of Foxp3^+^ Tregs in spleen from NOD mice treated with RGI-3100-iB (data not shown), RGI-3100-iB can modify the function of Tregs that infiltrate islets in an antigen-specific manner and might suppress islet-infiltrating pathogenic cells through the bystander suppression effect [11, 12].

In this study, RGI-3100-iB treatment was initiated at 3 to 4 weeks of age in NOD mice. The ages of NOD mice are equivalent to prestage 1 in humans, at which time individuals carrying T1D susceptibility alleles have not yet developed islet autoantibodies and exhibit no signs of active autoimmunity [37]. Moreover, an immunotherapy at this stage is much safer. As evident from clinical trials as well as rodent experiments on an insulin-based immunotherapy, the treatment should be initiated in the early phase of T1D onset or in prediabetes, because the established effector memory pathogenic T cell responses in the late stage of T1D are not curbed by the antigen-specific monotherapy [5, 6]. A liposomal *α*-GalCer (RGI-2001) has previously been shown to be safe in human clinical trials [38], and because RGI-3100-iB has the same liposomal formulation, it might be a possible candidate for testing in the prevention of T1D. On the other hand, compared with prestage 1 trial, stage 1 clinical trial is more acceptable, where individuals have developed measurable signs of autoimmunity in the form of autoantibodies, and there are indications that very limited beta cell loss occurs prior to diagnosis [39]. In the NOD mouse study, 9–10 weeks of age is equivalent to stage 1, the age wherein treatment should be initiated in NOD mice even long after the onset of insulitis. Interestingly, the high-dose treatment of aqueous *α*-GalCer in NOD mice starting at 10 weeks of age (5 *μ*g/mouse and booster at 11 and 14 weeks of age) showed significant protection of T1D in NOD mice [23]. Because our data indicated that RGI-3100-iB has more potent prevention efficacy than aqueous *α*-GalCer, it is worth examining whether RGI-3100-iB protects against T1D in NOD mice 9–10 weeks old. In addition, considering the fact that highly effective protocols in preclinical phase are not reproduced in clinical trials, the further enhancement of efficacy would be an important issue. One of the strategies for enhancing the efficacy is the combination of RGI-3100-iB with sirolimus treatment. In our clinical trial and in a rodent experiment, liposomal *α*-GalCer (RGI-2001) exhibited a trend of synergy with sirolimus (rapamycin) to promote Treg expansion and decreased the acute graft-versus-host disease after allogeneic bone marrow transplantation in patients [38, 40].

The question that needs to be answered is whether the loading of one epitope peptide rather than the entire protein onto the *α*-GalCer liposomes is sufficient for effective prevention. In this study, the insulin B 9–23 peptide was chosen as the target autoantigenic epitope to induce immune tolerance using our liposome technology because insulin is a prime candidate autoantigen in T1D and T cell responses to insulin B 9–23 are essential for the development of T1D in both humans and mice [41, 42]. We postulate that loading one autoantigen epitope is enough for the efficacy of our approach because of the properties of antigen-specific Tregs, namely, antigen-dependent homing and bystander suppression with TGF-*β* secretion [11, 43]. Moreover, using an epitope peptide has the following three advantages in terms of liposome loading: (1) proven clinical safety in an antigen-specific immunotherapy; (2) possibly easier manufacturing; and (3) reducing possible adverse effects generated by unnecessary epitope presentation [44, 45]. On the other hand, considering the heterogeneity of T1D in humans, a mixture of peptides consisting of reliable epitope candidates from several autoantigens could be worth exploring and might benefit patients with T1D [46–48].

## 5. Conclusions

In this study, we prepared liposomal *α*-GalCer, encapsulating insulin B 9–23 epitope peptide (RGI-3100-iB), with the aim of establishing an antigen-specific immune tolerance induction therapy for T1D. RGI-3100-iB prevents T1D onset in NOD mice, diminishes insulitis together with recruitment of Foxp3-positive regulatory T cells to islets, and enhances protection of regulatory cells against diabetogenic cells in an antigen-dependent manner. Because autoantigen-specific Treg cells are promising for the therapy of T1D, our results provide a potential candidate for an alternative therapy of T1D.

## Data Availability

The datasets generated during and/or analyzed during the current study are available from the corresponding author upon request.
